# Parents’ lived experiences of parental needs for support at a burn centre

**DOI:** 10.1080/17482631.2020.1855749

**Published:** 2021-01-11

**Authors:** Lina S. T Lernevall, A. L. Moi, E. Gjengedal, P. Dreyer

**Affiliations:** a Department of Plastic, Hand and Reconstructive Surgery, National Burn Centre, Haukeland University Hospital, Bergen, Norway; b Department of Global Public Health and Primary Care, University of Bergen, Bergen, Norway; c Department of Health and Caring Sciences, Western Norway University of Applied Sciences, Bergen, Norway; d Department of Public Health, Section of Nursing, Aarhus University, Aarhus C, Denmark

**Keywords:** Burns, child, interview, intensive care units, human needs, paediatric, parents, phenomenological hermeneutics, psychosocial, Ricoeur

## Abstract

**Purpose**: A burn injury to a child is a traumatic event and the parent’s emotional reactions and coping strategies affect the child’s adaptive outcome. It is therefore important that parents get the right support. The aim was to explore parents’ lived experiences of their need for support when having a child admitted to a burn centre.

**Methods**: Semi-structured face-to-face interviews were conducted with 22 parents of children age <12 years hospitalised with an accidental burn injury, 9 to 27 days after the burn accident, from April 2017 to July 2018. A Ricoeur-inspired textual analysis method was used.

**Results**: Four themes emerged from the analysis and describe the parents’ needs for support. The parents wanted to be taken care of as a whole family and feel safe in the hands of professionals. This, in turn, depended on being informed about the child’s condition and treatment, but also on getting help in dealing with feelings of guilt. Not least, parents wanted opportunities to take care of their own fundamental needs in terms of hygiene, food, adequate rest and activities.

**Conclusion**: As an overall understanding the healthcare providers should focus on the family as a whole in care and treatment.

## 1. Introduction

Childhood burns are among the most severe forms of injury, with high morbidity and mortality rates worldwide (Kim et al., 2012). In Europe, 50–80% of all burns in children affect children under the age of five (Brusselaers et al., 2010). In Norway, children between zero and three years of age are 12 times more prone to experience a burn injury than children >5 years and adults (Onarheim et al., 2014). Both the injury and the burn treatment are known to be painful. Treatment often includes reconstructive surgery and a long rehabilitation period (Greenhalgh, 2019; Wiechman & Patterson, 2004). Apart from the impact of the injury and its treatment, parents’ reaction and responses to the traumatic event have been shown significantly to influence the child’s functioning, wellbeing and phycological recovery (Bakker et al., 2013; De Young et al., 2014; Lieberman, 2004). Supporting parents of burn-injured children is therefore an important part of the regular care offered at burn centres.

Parents, mostly the mother (26%) or both parents (12%), are often present when a burn injury occurs, and the accident typically happens at home in the kitchen or dining room (47%) or the bathroom (12%) (Pardo et al., 2008). Parents can therefore easily be affected by negative feelings and emotions. A recent systematic review reported that many parents were affected by guilt, shame and blame (Kornhaber et al., 2018). Moreover, many parents developed anxiety reactions and traumatic stress due to their child’s burn injury (Bakker et al., 2013; Hawkins et al., 2019). For most parents, traumatic stress symptoms decline after the first months; in some, though, symptoms may persist for years (Bakker et al., 2013; M. Egberts et al., 2017; M. R. Egberts et al., 2018b). It has also been reported that mothers of burn-injured children have a higher depression score than the general population (El Hamaoui et al., 2006). When parents are affected by accident-related psychological distress, they are more challenged when seeking to positively support their child through medical care (Brown et al., 2019). A high state of parental anxiety combined with ineffective parental coping strategies can also cause the child to have a non-adaptive outcome after the burn injury (Simons et al., 2010). Hence, having a healthy and supportive family seems to positively affect the child’s health-related quality of life (Landolt et al., 2002).

A recent review of parental needs for support while hospitalised in a burn centre identified only seven articles, highlighting the sparsity of knowledge within the field (Lernevall et al., 2019a). The described support offered to parents included different kinds of group consultation (Barnett et al., 2017; Cahners, 1979; Frenkel, 2008; Rivlin et al., 1986), an internet-based information and support programme (Sveen et al., 2017) and parental presence during wound care procedures (M. R. Egberts et al., 2018a). Parents participating in group consultations found it beneficial to process and share their feelings and learn about coping strategies such as how to manage stress and how to maintain psychosocial wellbeing. The internet-based information and support programme was perceived as informative, comprehensive, meaningful and supportive, though some parents found it time-consuming. Regarding parental presence during wound care procedures, some parents were glad to have been present while others were happy they were not. A critical factor for parents’ choice was their emotional state prior to the wound care. A new study investigated how staff members at a burn centre supported parents of burn-injured children; the staff created a safe, secure and trusting environment upon arrival, addressed parental guilt, supported parents in doing daily routines and involved them in wound treatment before discharge (Lernevall et al., 2019b). This study did not, however, investigate parents’ actual experience and needs (Lernevall et al., 2019b). Assessing parents’ emotional status and support needs during their child’s hospitalisation may be difficult as they are so concerned about their child’s wellbeing that they supress their own needs, which also makes it difficult for them to receive psychosocial support (Griffiths, 2017; Heath et al., 2018). Parent-perceived isolation and barriers to psychosocial support as well as the current lack of evidence-based parental support programmes testify to the need for further research in this field (Heath et al., 2018; Lernevall et al., 2019b). The review by Lernevall et al. presents different types of support offered to parents of burn-injured children, but does not mention which kinds of support they needed and requested. The aim of the present study was therefore to explore parents’ lived experiences of their need for support when having a child admitted to a burn centre.

## 2. Materials and methods

### 2.1. Study design

This study is one stage of a multi-phase study investigating parents’ needs for support while having a child hospitalised with a burn injury (Lernevall et al., 2019a, 2019b). It is an explorative study that uses a phenomenological-hermeneutic approach (Dreyer & Pedersen, 2009) to gain a deeper understanding of the parents’ lived experiences. Phenomenology helps the researcher to look at how the world is experienced by the subject by studying different aspects of consciousness and experience (Zahavi, 2019). “ To get a deeper understanding of the parents’ lived experiences, a textual analysis method inspired by the French philosopher Paul Ricoeur was chosen (Dreyer & Pedersen, 2009). When recorded interviews with parents are transcribed, their lived experiences are transformed into a text. “The others mind’s experiences” are captured and maintained in the text (Ricoeur, 1976, p. 73). The text can then be analysed to understand the parents’ need for support during hospitalisation. To “grasp” the meaning of the text, one needs to use interpretation. Ricoeur argues that as soon as a text has left the author, its original addressee is released, and “A text is addressed to anyone who can read” (Ricoeur, 1976, p. 92). It is thereby possible for everyone to interpret the meaning in the text. This interpretation process is circular as it moves from looking at the parts in the text, then looking at the whole of the text, then returning to the parts again. In this way, one gets a deeper understanding of both parts and whole. During this process, one discovers “the sense of the text” (Ricoeur, 1976, p. 87) as the depth of the text is unfolded. To grasp the parents’ experiences of their needs for support, face-to-face interviews were used for data collection.

### 2.2. Setting

The study took place at a Norwegian burn centre with eight beds, five of which are burn-intensive care unit beds. The burn centre treats about 150 inpatients per year, of whom one third are childr,en. Patients are treated by a multidisciplinary burn team consisting of surgeons, nurses, anaesthesiologists, physiotherapists, psychologists, social workers, hospital clowns, psychiatrists and priests.

### 2.3. Participants and recruitment

Participants were parents (n = 22) of children (4 girls and 9 boys, mean age 2 years and 2 months (2 months to 7 years)) hospitalised due to an accidental burn injury. The participants were 12 mothers, 9 fathers and 1 stepfather, their mean age was 33 years and 3 months (21 to 46 years). Eighteen parents were together/married, three were divorced living alone and the stepfather was a partner to one of the divorced mothers. Eight parents had one child, while 14 had more than one. All parents were employed. Sixteen parents were Norwegian, and six were from other European countries. Nearly all spoke Norwegian fluently, except for two parents who mixed Norwegian and English, and got help from their spouses for the interview. Nine children had been burned with hot coffee, tea, soup or water; one had been burned with fire, one with electricity; and two had come into contact with a hot surface. Two injuries resulted in amputation, three children got skin graft surgery, seven would get a scar and five had a life-threatening but non-fatal injury. Some days after admission, all parents were seen by a psychologist, who recruited parents for this study. If the inclusion criteria were fulfilled (Table I), parents were informed about the study and invited to participate. Thirty invitations were handed out (27 in Norwegian and 3 in English). The first author, who is a former burn care nurse, contacted all parents who consented to participate, and dates were set for an interview.

**Table I.** Inclusion criteria

– Parents who could speak and understand Norwegian, need not be ethnic Norwegians. During the inclusion period, this criterion was changed to include all parents (no restrictions on language) due to low participation. If parents could not speak Norwegian, an interpreter would be present at the interview.
– The child had to be under the age of 12 years. The Norwegian “Barnelova” (The children’s law) § 31 states that a child who has turned 12 years must say its opinion about decisions regarding personal matters (Barnelova, 1981). If the child were to be 12 years or above, the child would also need to be interviewed which would change the aim of this study.
– The children and parents had to be hospitalised for a minimum 24 hours to have the experience of being at the ward.
– The burn injury had to be caused by an accident. Any burn injuries caused deliberately should not be included.

### 2.4. Data collection

The first author conducted all face-to-face interviews (Table II) from April 2017 to July 2018. Sixteen interviews were completed. There was no need for an interpreter as parents managed to express themselves. Parents decided themselves whether they wanted to be interviewed together (n = 12 parents) or separately (n = 10 parents) and where the interview should take place. All parents appreciated, and some were even thankful, for the opportunity to participate in this study and share their experiences. The semi-structured interview guide used had been tested on four parents who were part of user involvement in research (these interviews were not included in the analysis). The main topics in the interview guide were how the parent(s) experienced being at the burn centre, how they experienced the staff members, what was meaningful to them and if they had been or missed being supported. The interviews were digitally recorded and transcribed verbatim, 14 by the first author and 2 by a secretary.

**Table II.** Characteristics regarding the interviews (n = 16)

Duration of interviews: Mean (min-max)	Total: 20 hours, 12 minutes and 15 seconds Mean: 1 hour, 15 minutes and 46 seconds. (33 minutes—2 hours and 55 minutes)
The number of days since burn injury when interviewed: Mean (min-max)	17 days (9 days—27 days)
Place of interview:	At the hospital near the burn centre (n = 8), at a hotel (n = 2), at a local hospital (n = 1) or in parents’ homes (n = 5).
Hospitalized or discharges:	Hospitalised (n = 8) and discharged (n = 8).
Interviewed together or individually:	Interviewed together (n = 6 interviews) and interviewed individually (n = 10 interviews) by the choice of the parents and depending on the situation (were both parents at the hospital together, was one at home or were they divorced?)

### 2.5. Data analysis

Data were managed in NVivo 12 Plus (QSR International Pty Ltd., 2019). The transcripts were read and analysed by the first and last author, using a Ricoeur-inspired method (Dreyer & Pedersen, 2009). An in-depth analysis, which resulted in a comprehensive understanding of the lived experience, brought the researchers closer to the parents “being in the world” (Dreyer & Pedersen, 2009, p. 65). The method consists of three steps: a naïve reading, a structural analysis and, a critical analysis and discussion.

The first step is a naïve reading, where the whole text is read so the researcher gets an “immediate understanding of the meaning content” (Dreyer & Pedersen, 2009, p. 67). In our study, all interviews were read as one text and a short narration (an A-4 page) was created from this first impression of the text as a whole (Table III). This was done to show the first analytical process.

**Table III.** Naïve reading (short version)

In the traumatic chaos following an acute accidental burn injury to a child, being transferred to a burn centre, the parents are somewhat calmed by knowing that they will be treated by the country’s top specialists within burn care; but they are also frightened, realising the seriousness of the situation. Arriving at the burn centre, parents meet staff members who are waiting ready to start treatment immediately. This first meeting is particularly important for the parents as it creates a trustful and safe atmosphere. However, parents are frustrated when they cannot get specific information about how it will all turn out or how long they have to stay. When being transferred to the burn centre, both parents want to travel together; however, this is not always possible. Parents arriving alone long to hear news about their child, but are surprised to realise that the door to the department is locked. They have to ring a bel for someone to come and open up. Again, they are surprised to be questioned about who they are and to learn that they cannot sleep at the burn centre. Many fathers explain how important it is for them to be there for their wives and support them. Therefore, they find it difficult that they cannot stay together as a family. Many parents have feelings of guilt, and they feel that staff help them deal with this guilt. It added to parents’ stress level and workload when they experienced miscommunication among staff members and lack of a contact person with an overview. The most important thing for parents was that their child was treated. However, expressed very modestly, parents also wished that some of their own fundamental needs would be met. They wished to have access to a shower so that they could clean themselves and to get a break so that they could think for themselves for just some minutes.

The second step is a structural analysis, consisting of three steps: 1) meaning-bearing units are found “What is said in the text?”, 2) significance-bearing units are found/created “What does the text talk about” and 3) themes are created (Dreyer & Pedersen, 2009) (Table IV). In our study, we started to look across the data for sections or paragraphs to understand “What is said in the text”. Sections were highlighted and then grouped. Each group was then examined for “What does the text talk about”. A narration using poetic language was generated, creating a distance in the interpretation, where we as researchers became more distanced from the text. As the third step, the narrations were given names or themes, which allows the researcher to become even more distanced from the text. This process is not straightforward. Instead, it is a circular movement between the three steps to ensure that the meaning is not changed or the parents’ words are lost. Throughout the structural analysis, there is a constant movement between explanation and comprehension, where one understands the whole by understanding the parts and so on, forth and back (Dreyer & Pedersen, 2009, p. 68).

**Table IV.** An example of the structural analysis of “3.1. Being in it together—for their child”

Meaning-bearing units (What is said in the text?)	Significance-bearing units (What does the text talk about?)	Theme
“It’s obvious that I would have had him here all the way. That’s obvious. But then they haven’t arranged it so that we can be the two of us. He didn’t get to stay with us. […] but for my part, then it would have been a huge support if he had also been allowed to be there (M1)”. “The first day it was quite nice to be two, so we had a chance to talk about it together and things like that (F3)”. “I don’t know how other parents are, but we … we complement each other. […] While I’m at 100, he stays at 50, and then we end up at 75 when there are the two of us (M12)” “He (red. husband) has been a wingman … (M14)”. “It costs us a lot to order tickets and everything, but now it’s important that we are together as a family (M16)”. “I think it would have done something if the family could have been two on the room some nights […] a family would appreciate to be together (M17)”. “But then again, the ideal solution would be for me to sleep here (red. at the burn centre). That would have been normal in a perfect world (F18) “. “I didn’t want to sleep because I wanted to be with my son … all the time. So, we changed like; he (red. husband) slept a few hours and after that we changed (M19)”. “And I think it’s much better for us, ehh, if we stay together the whole time. And then the doctor said ‘Okay today you can, you can stay and sleep here’, right. And both of us smiled and that was so nice, right (F20)”. ““I saw that she was in shock. She was so sad that she burned X (red. the child) and I knew that I had to be near her. […] I wasn’t allowed to stay. I was only allowed to visit. Nothing more. But we wanted to be all of us together (F22)”. … (there are more citations).	… The couples described themselves as a team complementing one another and therefore wanted to be together during the traumatic and chaotic situation they were in. The couples tried to help each other and stay positive during the difficult times. This was a way of taking care of each other and themselves. Being together was described as very comfortable, safe, helpful and nice, both during the day and during the night. When they experienced the same things, they could more easily share the burden; and, for example, at night being only the two of them, they went through what had happened. In that way, being together was described as extremely valuable to the processing of everything that had happened. Even physically separated couples called each other using video-calls, because having each other made it easier to cope, and some said that their relationship became stronger. For various organizational reasons, both parents were seldom allowed to spend the night at the burn centre. …	Being in it together—for their child

As a final and third stage, the findings from the structural analysis were critically analysed and discussed by all authors in light of other relevant literature, research studies and theory. Through the critical analysis and discussion, we got an in-depth understanding of the interpreted themes: the parents’ needs for support. This third step is integrated in the discussion.

### 2.6. Ethics

This study followed the Helsinki Declaration (World Medical Association, 1964/2013) and was ethically approved by REC—the Norwegian Regional Committees for Medical and Health Research Ethics (REC, 2019), project number: 2017/54/REK. Informed consent was obtained from all parents, and no one from the burn centre knew who participated. All audio records were digitally recorded and kept in a secured research server at the university hospital. All names of individuals and places were removed to anonymize the transcripts.

All parents were interviewed by the first author, who was familiar with the burn centre but not with the participants. She was particularly observant of parental reactions during the interviews to ensure that parents could be followed up if needed by a psychologist. Parents who got strong emotions during the interviews were asked if they wanted to stop the interview (all wanted to continue) and if they needed to talk to a psychologist (all declined).

Halfway through three of the interviews, the first author realized that five parents had been wrongfully included which represented an ethical problem. To respect the parents, their shared experiences and time used, the interviews were continued and data from these interviews were included for further analysis.

## 3. Results

Four themes showing the parents’ needs for support were found during the analysis. These themes will be presented in the following text.

### 3.1. Being in it together—for their child

An accidental burn injury in a child was experienced as a traumatic event by the parents. They dealt with the burn accident together, one way or another. In most cases, both travelled to the hospital together; some drove while others were transported by air ambulance or helicopter. Couples wanted to travel together; however, it was not always possible due to lack of space in the helicopter.

The couples described themselves as a team complementing one another and therefore wanted to be together during the traumatic and chaotic situation. “I don’t know how other parents are, but we … we complement each other. […] While I’m at 100, he stays at 50, and then we end up at 75 when there are the two of us (M12)”. The couples tried to help each other and stay positive during the difficult times. This was a way of taking care of each other and themselves. Being together was described as very comfortable, safe, helpful and nice, both during the day and during the night. When they experienced the same things, they could more easily share the burden; and, for example, at night being only the two of them, they went through what had happened. “The first day it was quite nice to be two, so we had a chance to talk about it together and things like that (F3)”. In that way, being together was described as extremely valuable to the processing of everything that had happened. Even physically separated couples called each other using video-calls, because having each other made it easier to cope, and some said that their relationship became stronger.

For various organisational reasons, both parents were seldom allowed to spend the night at the burn centre. Many parents were surprised about this and found it stressful and a hassle to find other accommodation for one parent. For some families, this extra expense was too costly, and only one could stay. However, most families payed so that they could handle the situation as a family, coming through it together. One mother explained the scenario if they would have been forced to choose that only one of them stayed:

“ … for his sake (red. the child), then dad would have travelled. Because he is more mentally stable so that he can handle and process … […] I’m more … mother, 100% mother. And I don’t think about anything else than X (red. the child). Ehh, I would have been sitting at home and probably been completely … crazy (M12)”.

Most often it was the father who slept elsewhere, for instance, in a nearby hotel or with relatives. The fathers said it was unpleasant not being hospitalised with the mother and the child. “It was quite sad to feel a little unneeded (F8)”. The fathers wanted to be support persons who were present and could help, also during the night. One father was described as a wingman; one who was there on the side, but present to step in when needed. Some fathers explained that it would be better for both of them to stay together because they were not so easily stressed as their wives. “I saw that she was in shock. She was so sad that X (red. the child) got burned and I knew that I had to be near her (F22)”. Being together, they could also relieve each other by taking turns. One parent was so afraid to lose her child that she could only sleep with her husband present.

Sometimes, one parent had to leave to go home for various reasons such as tending to other children, work, getting extra clothes, etc. Being alone at the burn centre was experienced as stressful, energy-consuming, sad, tough and as putting extra pressure on the one parent who stayed, especially during the first days, the acute phase. They were longing for their partner and found it hard to deal with everything on their own.

Parents with more than one child felt divided between their need to care for the hospitalised child and for their child/children at home. In some families, the siblings visited the hospital. But for some it was too expensive, and they communicated using video-calls from home. It burdened the parents not to meet their other child/children during the hospitalisation.

The parents wanted to be there for their child; even parents who were divorced. They were also in it together as parents to the same child, but had no desire to be at the burn centre together with the other parent. Divorced parents with less good contact acknowledged that the other parent needed to be around the child, but were exhausted to be around the other parent the whole day, every day. For some, the situation was even more challenging when the staff mistakenly thought that they were still married and treated them as a couple. This made the parents irritated, frustrated and unsure if the staff knew about their situation.

### 3.2. Being taken care of by professionals makes you feel calm and safe

Upon arrival at the burn centre, many parents described themselves as terrified, alert, sleep-deprived, exhausted, stressed and filled with fear. However, as soon as they were finally there, they relaxed, knowing they were at last with burn specialists. The parents were really moved seeing so many people waiting for them and they felt prioritised and taken care of.

“ … we were greeted by a whole team up there who knew that we were coming. They had made everything ready to start treatment on her (red. the child) immediately. You felt in a way very safe at once you entered the department (F4)”.

The way the parents were met upon arrival was highlighted as particularly important and described as “accommodating” or ‘being cared for”. They felt met and seen as staff greeted them in an open way, introduced themselves and told what they were going to do. Parents felt comfortable, relaxed and cared for when staff listened to them, answered their questions and were always patient with them. They experienced staff members as trustful, helpful, self-confident, skilled, calm and caring, all of which made them trust the staff to care for their child. “I’ve said to everyone that it’s angels in hospital coats that work here (laughs) (M16)”. What the parents experienced as particularly important was that staff were there for them no matter how busy the department was. “And you feel that they have enough time to answer, that it isn’t just a production line (M1)”. Having or taking the time to listen and answer questions was of importance to the parents.

Some parents were reassured by the staff that things would be fine even though they were not promised anything by the staff. Others calmed down as they saw how medical equipment was removed from their child such as a respirator, intravenous fluids, urine catheter and other cables. They were happy to hear that the child did not need it anymore and reassured that it might not be so bad after all. “And it was very nice when I came in: they had removed it all, catheter and surveillance and cables … except that they had given her a feeding tube. And when I came in, my wife was standing holding her in her arms without all the cables. That meant a lot (F8)”.

When fathers did not arrive together with their child, they were very happy and emotional finally to arrive, longing to hear news about their child. When reaching the burn centre, they were astonished to realize that they had come to a locked door where they had to ring a bell for someone to lock them in and on top of that hear: “It’s not possible for you to sleep here. There is only room for one relative (F18)”. After all, they were happy to be reunited with their family; but at the same time, it was experienced as shocking and frightening to enter a room full of unfamiliar people without knowing what was happening or how the child was doing.

Nearly all parents had feelings of guilt upon arrival, including those who were not present when the accident happened. “No matter what, you feel guilty for not being able to protect your child, which is the most important task you have. And then it is the most vulnerable person, the youngest person in the family who gets to suffer the worst consequences (M16)”. The way staff members reacted and approached their feelings of guilt meant much to the parents. Staff members told them that they should not blame themselves or ruminate about it; that it was not their fault; that they were not the only one who had experienced that their child got burned. “I really felt that I had done something terribly wrong. The doctor from the emergency department, he really placed all the guilt on me. And that was the first thing they said at the burn centre: ‘These things happen. And it can happen to anybody’ (M10)”. Some parents, though, got puzzled and felt uneased if staff raised the topic suddenly and without context; but after a while, they accepted that doing so had had a helping effect. However, not all parents were open enough to share their feelings, and they suffered alone. Even though family members tried to address the topic, it made a difference hearing it from the staff. “Someone that really tells you: ‘It’s normal to have these feelings, and in time they will … disappear. It will not entirely disappear forever, but … it will get easier in time … and then it actually isn’t your fault’ (M12)”. Furthermore, it helped to talk to other parents at the department.

### 3.3. Trying to have some control in an uncertain situation

The parents got really frustrated when they did not know what was going on or what to expect next. In their chaotic and uncertain situation, they wished to have concrete information to hold on to. Being informed made them understand what was happening, made them more optimistic and unworried and not so afraid of bad news. “But here we get to talk to the doctor straight away, and that’s really great. It makes it much safer as a mother (M14)”. When they experienced that staff were honest with them, whether they delivered positive or less positive information, they trusted them more and felt safe. Parents who had received a “Welcome to the burn centre”-pamphlet containing information about the department’s routines when they arrived felt calmer and better prepared for what to expect. This made it easier to ask the doctor about things they wondered about or did not understand. Divorced parent with less good contact did not share all information, resulting in the fathers still being stressed and lacking information. A stepfather felt that his presence and existence went unrecognised, as he was not a legal parent of the child. Many parents felt that there was a lack of structure, and they felt alone and had to spend enormous amount of energy trying to get an overview of the situation. Many experienced that staff members gave contradicting information, which made them confused, irritated, despairing and hampered their ability to navigate the information given. They wished for some standard information about the department but also information about burn injuries, and some searched the Internet but were unsure which information to trust. Not getting any information after wound treatment or surgery made parents impatient, frustrated and scared that something was wrong.

“There was very little information. Both before and after wound treatment and when they transplanted some skin and we were at the recovery and we were there to pick him up. I talked to the anaesthesiologist … he wouldn’t say anything because he wanted the surgeon to say it. But the surgeon didn’t come (F9)”.

Upon arrival as well as during hospitalisation, they wanted to know when they were being discharged. Getting contradicting information about this, parents became scared that the situation might be worse than they had thought and unsure whether the staff were withholding information from them. One couple had mistakenly been told that they had to stay for another 12 days, just to find out that they got discharged the following day. This made them distrust the staff; and when staff could give them no specific departure date, parents got frustrated, disappointed, sad, unsure or angry. “And I asked ‘Well how long do we have to stay here?’. ‘Yes at least 10 days more, because 20 days is standard procedure’. […] well I feel like I’m never coming home, because they say so many different things (M10)”. When staff members told them about the process of wound healing, they could more easily relax and accept the uncertainty of departure even though they got irritated. “Even though it makes it difficult to plan, it would have been nice to know (red. when to go home), yes (M1)”.

Some parents, though, were better at taking one day at a time, whereas not knowing really affected other parents. Being informed about the time of discharge and future caring tasks at home made them more relaxed, calm, safe and secure of their role, and gave them time to think everything through, prepare questions and focus on the tasks ahead. However, receiving this information on the day of discharged was experienced as extremely stressful. Even though they were happy to hear that they could finally go home, abruptness of discharge made them unsure what was going on or if they had been forgotten. Many also forgot to ask questions and to get all the papers they needed, even though they had both questions and worries.

“Maybe they could have had some kind of end-conversation or recap. […] Because my version might quite certainly be different from the staff members’ version, just so that we, in a way are on the same page (M15)”.

### 3.4. Getting time to be yourself and see to personal needs

Although parents were happy to be at the burn centre, they found that being hospitalised was a strenuous experience. The burn centre was not like other hospital departments; parents described it as being isolated and with very strict hygienic rules. “One door, two doors, a lot of disinfectants, on with clean cloaks, off with that and on with that (breathes heavily). Ahhh … help (M12)”. In the first acute phase, they appreciated all the care and the kindness when they were being brought things; but after a while, they became a little passive. The parents had to adapt to the rules and routines of the department, which some of them found really hard. “And THEY are the one who must get us some food […]. And we’re not … helpless. We can manage OURSELVES. ‘Well, then, we better follow their schedule’! I think it’s very much, it’s like being in a prison (M17)”. They wished to do everyday chores while hospitalised, a way to gather energy. One couple changed weekly, as one stayed at the hospital and the other at home. “It has been so nice for me to be at home with our daughter and then come back with recharged batteries (M16)”.

The parents longed to get just a small break, for instance, to get a cup of coffee and think about something else, but it was hard to ask for it themselves. “Not like they had to take the child for hours, but just 10 minutes here and there if one had to some small errand (M15)”. They did not want to be a burden, to be perceived as too demanding when asking for time alone. However, only few parents experienced that staff could babysit the child to give them a small break. “She asked, ‘Is there anything I can do for you’? And I felt like screaming (red. gets a wobbly voice and almost cries). But I didn’t, I kept in within me. She was the first one in 16 days who had asked me (M17)”. The parents highlighted how important it was for them to get out, and, for instance, exercise so that they could let go of their feelings.

When parents were offered personal time to eat, drink and maintain personal hygiene, they were really touched and felt treated with dignity and as a human being. Getting time to eat while the child was undergoing wound treatment in anaesthesia or taking a shower was also extremely important. Only few parents were offered to use the staff shower in the hallway, as there were none in the patient’s rooms.

“I REALLY missed to be offered a shower. I was CERTAINLY not clean all those days; […] I didn’t feel that I could, I couldn’t leave him, […]. But what I did was, while he was being operated, then I washed my hair in the sink (laughs). I was quite desperate (laughs) (M15)”.

The parents were happy that staff offered to wash their clothes in the department’s washing machine, especially when acute transferred to the burn centre.

The days at the burn centre were experienced as “very long (M1, F8, F13, M15, M16)”, and the parents tackled this differently. Some had brought books, computer, mobile phones or kept a diary. Others relaxed in the patients’ living room at the burn centre. Those who had a television on the room were happy to be entertained. Meeting the hospital clowns was also a possibility to get a pause from everything. “It’s not something that takes a long long time, but all of a sudden, for 15 minutes, you forget that you’re at the hospital (M12)”. Parents talking about the clowns smiled and laughed as they were retelling what the clowns did. The hospital clowns gave them a positive experience.

When someone had time to sit down and drink a cup of coffee together with them, talking about everything and nothing, they felt that others took interested in their life and in them as a person. One way of getting a chance to talk things through was seeing the psychologist or a social worker. Many parents had also contacted a psychologist in their hometown. Some mothers staying alone at the burn centre got help from their own mother who came and stayed nearby the hospital. Having their mothers there was experienced as a kind of self-therapy.

### 3.5. Overall comprehensive understanding

All four themes create a comprehensive understanding showing the parents’ fundamental needs to be seen and treated as a unique individual. Parents need to be together with their partner during a traumatic experience, they need to feel safe in the hands of professionals, and they try to cope in an uncertain situation and to see to their own fundamental needs.

## 4. Discussion

Our results show that parents’ need for support was very much an existential need. In the face of the difficult situation following a paediatric burn injury, they wanted to be taken care of as a whole family and feel safe in the hands of professionals in order to be there for their child. This, in turn, was closely linked to being informed about the child’s condition and treatment and about routines and future prospects. Not least, parents wanted the opportunity to take care of their own fundamental needs in terms of hygiene, food, adequate rest and activities.

We found that being together to support each other was a prime need for couples of burn-injured children. The traumatic situation of having a burn-injured child was handled as a team by couples; and they had a strong need for being together both during the transfer to the burn centre and during their stay, day and night. Being separated was therefore hard and challenging. The need to face challenges as a team has also been reported in two other Australian and Indian studies of parents of burn-injured children (McGarry et al., 2015; Ravindran et al., 2013b), implying that this is fundamental to parents rather than a culturally determined need. Our data also show that divorced parents had other needs than couples. They still needed to be together with the child but not with the other parent. This particular need and the challenges involved in treating divorced parents seem not to have been discussed in the burn literature. However, previous studies of critical care settings have described that staff should be aware of divorced couples if major differences or conflicts still exist when decisions are made regarding the patient (Leon & Knapp, 2008). Our results highlight that being treated as a family strengthens parents, which is in line with family-centred care (FCC). FCC means caring for both the child and its parents, using the four concepts: “respect, dignity, information sharing, and participation and collaboration” (Foster et al., 2016, p. 432). Staff should have time to listen to and answer parents’ questions, as this study shows. Years ago, FCC was implemented as a philosophy within paediatric nursing (Harrison, 2010), and parents of hospitalised children have reported overall positive experience with FCC (Arabiat et al., 2018). This perspective should also be highly relevant for burn centres treating children. Parents should therefore be included in the care.

Parents with more than one child had a double responsibility as they had to care not only for their hospitalised child but also for the child/children at home. For some, this was difficult to balance in a good way, and not being able to be both places could add to their feelings of guilt. Staff members should be aware of the parents’ worries and support them to maintain contact with their family at home.

Our data suggest that parents need to talk not only about feelings of guilt, but also about how to deal with these feelings. In the present study, parents’ willingness to share their feelings and thoughts seemed to be linked to how safe they felt in the care of burn staff members. Parental feelings of guilt seem to be common in relation to paediatric burn injuries (Kornhaber et al., 2018; Sveen & Willebrand, 2018) and critically ill children (Engström et al., 2015). The present study shows that the way staff members approached the topic was pivotal. Hence, staff can either make parents feel worse by assigning guilt to them or make them feel better, helping them by telling them that accidents do happen. When parents are assigned guilt for their child’s injury, their belief in themselves as good parents weakens, as also reported in another study (Ravindran et al., 2013b). Staff members should recognise their influence on parents’ feelings of guilt.

Another main finding of the present study is that parents felt a strong need to gain some control by getting information. Getting information either from a written welcome pamphlet or by talking with the staff made parents feel calm, safe, less afraid, prepared and more trusting. Not being informed, getting contradicting information or not being answered, on the other hand, made them feel frustrated, angry, stressed, scared, impatient, irritated and despairing. Our findings here echo those of a study of parents to children in an intensive care unit in which parents felt calmer the more information they got; and more stressed, insecure and afraid when they received no information (Engström et al., 2015). A need for information shortly after wound treatment and operations, as well as being well prepared and informed about discharge, was an important finding in our study.

A study from 2008 investigating how to involve family systems in critical care nursing found that family stress lowered when information was provided continuously (Leon & Knapp, 2008). Information given in continuous, frequent and small portions was more easily absorbed by parents (Engström et al., 2015). The legal right to receive information differs if you are a parent or a stepparent. Knowing how modern family structures vary, we find that more emphasis should perhaps be devoted to stepparents’ information needs.

In the present study, getting time to see to one’s own fundamental needs such as having time to eat, drink, clean oneself, do some exercise, talk, laugh and have a break were essential for parental wellbeing. This can seem like a very natural thing, and maybe so natural and fundamental that it is easily forgotten or overlooked. It might be worth reminding ourselves of Maslow’s hierarchy of human needs, according to which basic physiological needs have to be fulfilled before catering for higher ranked needs like safety, love and belonging, esteem, and self-actualisation (Jackson et al., 2014; Mohammadhossini et al., 2019). Henderson and Orem have also described fundamental human needs, and they added a number of important aspects such as keeping the body clean and well-groomed; communicating by expressing feelings, needs, fear, etc.; playing or participating in different kinds of entertainment; and balancing between being alone and having social contact (Henderson, 1964; Orem, 1971). Our data showed that parents needed to talk to both staff members and other parents, which has also been described elsewhere (Engström et al., 2015; Heath et al., 2018). However, our data also displayed a parental need for some time alone to see to personal needs, which seems not to have been addressed in prior burns research. Our analysis revealed that a burn centre can give parents a feeling of being isolated or in prison. Other studies also found that being in the strict hygienic environment of an intensive care unit, the parents felt isolated and focused only on their child, making them neglect some of their basic needs (Foster et al., 2016; Heath et al., 2018).

### 4.1. Strengths and weaknesses

To ensure complete reporting of all relevant matters and to enhance trustworthiness and transparency, we used two guidelines for qualitative research (O’Brien et al., 2014; Tong et al., 2007). Trustworthiness and transparency were strengthened by using verbatim transcription, a meticulously described step-by-step analysis and by justifying the findings using citations with the parents’ own words.

Another important strength of the present study is that 45% (n = 10) of the parents were fathers or father figures. Even though the mother is considered the main caregiver in some cultures (Ravindran et al., 2013a), it is important to take the fathers’ perspectives into account. Parents were included consecutively, and by pure chance they displayed much variety, for example, in terms of nationality, sex, parental role and length of stay. This diversity is seen as a further strength. Purposeful sampling was not possible and is a shortcoming of the present study. However, as we included patients hospitalised at a burn centre; there is little doubt that all burn injuries were severe and that the parents had rich experiences to share. The divorced parents added knowledge about the need for equal treatment of parents, especially when communicating about sensitive issues, that the parents may find difficult to discuss between them. The stepfather also highlighted an unnoticed problem of how stepparents are met and treated. Further studies should investigate if these experiences were just a single case or a more general problem. Even though this study targeted parents of burn-injured children, some of their experiences and the study findings in general may be comparable to those of parents of children suffering from critical illness.

Five parents were wrongfully included as their stay lasted less than 24 hours. This challenged our preunderstanding, yet turned out positive as the parents were included in the study and contributed with important experiences that would otherwise not have been reflected in the material.

## 5. Conclusion

In this study, we explored parents’ lived experiences of their need for support when having a child admitted to a burn centre. In the context of facilitating their positive contribution to their child’s treatment and recovery while hospitalised at a burn centre, they had different needs for support. However, they all shared a need to be cared for as one whole family, including siblings at home, facing the situation as a team while supporting each other. At the same time, they needed support from the multidisciplinary burn team; they needed help in dealing with their feelings of guilt and they needed information to gain some control over the situation and to be informed about their child’s condition and treatment and about routines, discharge and future prospects. They also needed breaks during the day to see to their own fundamental needs in terms of hygiene, food, adequate rest and activities, and to recharge their batteries. Our study shows that it seems essential that healthcare providers focus on the family as a whole when a child is hospitalised and treated for a burn injury.

## 6. Implications

Based on this study, some advice can be given to burn centres treating children. A strategy on how to welcome, treat and discharge parents is needed. When possible, both parents should be transferred together and allowed to stay together both during the day and during the night. It should also be considered to assign a contact person to each family to ensure correct information; and to consider which kind of information to give and in which form. Further suggestions include ensuring some predictability and offering daily breaks where the staff look after the child to ensure that parents have time for personal hygiene and rest. Having hospital clowns at burn centres can offer relief, making parents momentarily laugh and forget about the situation. A multi-disciplinary approach is needed to support parents after a burn accident in their child.
